# Systematic Elaboration of the Pharmacological Targets and Potential Mechanisms of ZhiKe GanCao Decoction for Preventing and Delaying Intervertebral Disc Degeneration

**DOI:** 10.1155/2022/8786052

**Published:** 2022-04-22

**Authors:** Wanqing Sun, Yuan Chen, Miao Li

**Affiliations:** ^1^Third Intenal Department, Hunan Rehabilitation Hospital, Hunan, China; ^2^The Maternal and Child Health of Liu Yang, Hunan Province, China; ^3^Department of Pediatric Orthopedics, Hunan Children's Hospital, Changsha 410007, China; ^4^The School of Pediatrics, Hengyang Medical School, University of South China, Changsha 410007, China

## Abstract

**Background:**

ZhiKe GanCao Decoction (ZKGCD) is a commonly used traditional Chinese medicine in the clinical treatment of intervertebral disc degeneration (IDD). However, its active ingredients and mechanism of action remain unclear. This study aims to propose the systematic mechanism of ZKGCD action on IDD based on network pharmacology, molecular docking, and enrichment analysis.

**Methods:**

Firstly, the common target genes between ZKGCD and IDD were identified through relevant databases. Secondly, the protein-protein interaction (PPI) network of common genes was constructed and further analyzed to determine the core active ingredients and key genes. Thirdly, gene ontology (GO) and Kyoto Encyclopedia of Genes and Genomes (KEGG) enrichment analysis of common genes were performed. Finally, the stability of the binding between core active ingredients and key genes was verified by molecular docking analysis.

**Results:**

“Intersecting genes-active components” network consists of 154 active ingredients and 133 common genes. The ten key genes are AKT1, TNF, IL6, TP53, IL1B, JUN, CASP3, STAT3, MMP9, and MAPK3. Meanwhile, quercetin (Mol000098), luteolin (Mol000006), and kaempferol (Mol000422) are the most important core active ingredients. The main signal pathways selected by KEGG enrichment analysis includes AGE-RAGE signaling pathway in diabetic complications (hsa04933), TNF signaling pathway (hsa04668), IL-17 signaling pathway (hsa04657), cellular senescence (hsa04218), apoptosis (hsa04210), and PI3K-Akt signaling pathway (hsa04151), which are mainly involved in inflammation, apoptosis, senescence, and autophagy.

**Conclusion:**

This study provides a basis for further elucidating the mechanism of action of ZKGCD in the treatment of IDD and offers a new perspective on the conversion of the active ingredient in ZKGCD into new drugs for treating IDD.

## 1. Introduction

Low back pain (LBP) is one of the most common global health problems, and it has been younger in recent years [1]. According to the Global Burden of Diseases Survey, LBP ranks among the top causes of disability [2, 3]. Intervertebral disc degeneration (IDD) is the most significant factor in LBP patients (40%) [4]. The vast majority of studies have suggested that LBP caused by IDD may be closely related to local inflammatory, but the specific mechanism is not yet fully understood [5, 6]. Therefore, the current pretreatment of IDD is still based on a single symptomatic treatment, and the main clinical drugs used are nonsteroidal anti-inflammatory drugs (NSAIDs) [7, 8]. There is a lack of effective noninvasive methods to control, slow down, and reverse the progression of IDD.

Traditional Chinese medicine (TCM) has been used for thousands of years in Eastern countries and is widely used to treat various diseases [9, 10]. In recent years, an increasing number of scholars have advocated a novel treatment method combining TCM and Western medicine. ZhiKe GanCao Decoction (ZKGCD) is a classic empirical formula for the treatment of IDD, which was developed by Professor Gong Zhengfeng with his years of clinical experience [11]. Professor Gong Zhengfeng is one of China's leading traditional Chinese medicine practitioners and has received many national honors. ZKGCD is mainly composed of Aurantii Fructus (Pinyin: ZhiKe (ZK)), Licorice (Pinyin: GanCao (GC)), Angelicae Sinensis Radix (Pinyin: DangGui (DG)), Radix Salvia (DanShen (DS)), Sparganii rhizome (Pinyin: SanLeng (SL)), Curcumae rhizome (Ezhu (EZ)), Semen Pharbitidis (Pinyin: QianNiuZi (QNZ)) (Table 1). The main effect of this formula is to invigorate blood circulation and remove blood stasis, which can alleviate the patient's pain by improving the local microenvironment of the herniation and the nerve root [11]. Currently, ZKGCD is mainly used by TCM practitioners to treat cervical and lumbar spine disorders and skeletal muscle disorders associated with inflammation [12–15]. However, the specific mechanism of action has not been elucidated and verified. Then, TCM is characterized by multiple components, multiple targets, and multiple mechanisms, which limited the elucidation of specific therapeutic mechanisms.

The emergence of network pharmacology presents a new opportunity. Recent studies have confirmed that network pharmacology has good predictive performance in the study of different drug-disease interactions [16–19]. Network pharmacology is mainly used to explain the relationship among herbs, compounds, targets, signaling pathways, and diseases with a specific approach. Currently, network pharmacology has been successfully applied to study the relationship between herbal medicines and skeletal muscle diseases and to demonstrate the complex mechanisms of TCM diseases based on multiple compounds, multiple targets, and multiple pathways. In addition, gene ontology (GO) and Kyoto Encyclopedia of Genes and Genomes (KEGG) analysis combined with network pharmacology may provide more valuable and complementary information, thus further improving the predictive performance of potentially effective mechanisms.

**Figure 1:**
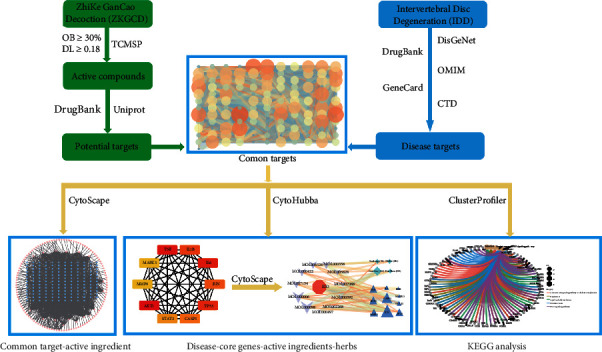
Flow chart of the study. OB: oral bioavailability. DL: drug-likeness.

This study aimed to explore the main active ingredients, potential targets, and signaling pathways of ZKGCD for the treatment of IDD based on the network pharmacology approach and to provide theoretical support for clinical practice. In addition, the reliability of the results was further confirmed by molecular docking.  Figure 1 illustrates the detailed workflow of this study.

## 2. Methods

### 2.1. Selection of Intersecting Genes for ZKGCD and IDD Target Genes

The formula of this study is ZKGCD, which contains seven herbs: Aurantii Fructus (Pinyin: ZhiKe (ZK)), Licorice (Pinyin: GanCao (GC)), Angelicae Sinensis Radix (Pinyin: DangGui (DG)), Radix salvia (DanShen (DS)), sparganii rhizome (Pinyin: SanLeng (SL)), curcumae rhizome (Ezhu(EZ)), semen pharbitidis (Pinyin: QianNiuZi (QNZ)). We used the Traditional Chinese Medicine Systems Pharmacology (TCMSP) database (https://tcmsp-e.com/) [20] to select the active ingredients of each herb in ZKGCD and set oral bioavailability (OB) ≥ 30% and drug-likeness (DL) ≥ 0.18. Meanwhile, the target genes corresponding to each active ingredient were obtained from the DrugBank database (https://go.drugbank.com/) [21] and UniProt database (https://www.UniProt.org) [22].

We integrated a total of 5 databases of target genes for IDD, namely Online Mendelian Inheritance in Man (OMIM) (https://omim.org/) [23], GeneCards database (https://www.genecards.org/) [24], Comparative Toxicogenomics Database (CTD) (http://ctdbase.org/) [25], DrugBank database (https://go.drugbank.com/) [21], and DisGeNet database (https://www.disgenet.org/) [26]. Finally, the obtained genes were uniformly named through the UniProt database.

The intersection gene set was obtained based on the above two gene sets by constructing Venn diagrams, which are potential target genes for ZKGCD treatment of IDD.

### 2.2. Network Construction and Core Gene Identification

In this study, we used Cytoscape software [27] to construct the “intersecting genes-active components” network and the “IDD-key genes-active ingredients-herbs” network to show the relationships among ZKGCD, seven herbs, intersection genes and IDD. In the network, the degree represents the number of edges shared by each node. Based on the degree of each active ingredient in the two networks, we used the top three active ingredients as the main active ingredients. Then, protein-protein interaction (PPI) network was obtained by importing all the intersecting genes into the STRING database (https://www.string-db.org/) [28] with *Homo sapiens* and 0.4 moderate confidence for filter conditions. At the same time, the TSV file was obtained. Finally, the obtained TSV file was imported into Cytoscape software to visualize the PPI network of intersected genes, and the PPI network was analyzed by MCODE and CytoHubba plugin in Cytoscape to acquire clusters and key genes with 12 kinds of topological measures.

### 2.3. Enrichment Analysis of Intersecting Genes

To further clarify the potential specific mechanism of the ZKGCD treatment IDD, we used the clusterProfiler package of *R* software to perform enrichment analysis for the intersection gene set, mainly including gene ontology (GO) and Kyoto Encyclopedia of Genes and Genomes (KEGG) enrichment analysis [29]. On the one hand, GO analysis was used to explore the potential relationship of intersecting genes with IDD treatment at three levels: cellular components (CCs), molecular functions (MFs), and biological processes (BPs). On the other hand, KEGG analysis reveals the main pathways of action of the target genes.

### 2.4. Verification of Stability for Core Genes and Active Ingredients

In this study, we mainly used molecular docking analysis to clarify whether there is a good stability between our selected key genes and the corresponding active ingredients. The stereo structures of key genes and active ingredients (small molecule ligands) were downloaded from the RCSB PDB database (https://www.rcsb.org/) and PubChem database (https://pubchem.ncbi.nlm.nih.gov/), respectively. And then, they were preprocessed by PyMol 2.4.0 and ChenBio3D software. Finally, we used AutoDock Vina to calculate the binding energy based on the hydrogenation reaction of proteins and small-molecule ligands.

## 3. Results

### 3.1. Bioactive Components and Drug Targets for ZKGCD

A total of 194 active ingredients from seven herbs in ZKGCD were obtained from the TCMSP database based on two selection conditions (OB ≥ 30% and DL ≥ 0.18). Among them, 5 active ingredients were derived from ZK, 92 from GC, 2 from DG, 65 from DS, 5 from SL, 3 from EZ, and 22 from QNZ (Supplementary Table 1). Then, 186 active ingredients were obtained after removing the repeats. Finally, 267 targets of ZKGCD were obtained by sorting the corresponding targets of 186 active compounds through DrugBank and UniProt databases (Supplementary Table 2).

### 3.2. Potential Target Genes of ZKGCD for the Treatment of IDD

**Figure 2:**
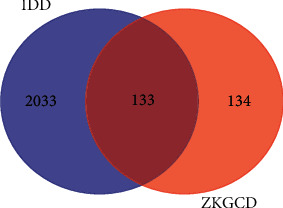
Venn diagrams of intersecting genes between IDD and active ingredients in ZKGCD. IDD: intervertebral disc degeneration. ZKGCD : ZhiKe GanCao Decoction.

We obtained a total of 2166 nonduplicated IDD-related genes from five databases OMIM, GeneCards, CTD, DrugBank, and DisGeNet database based on the keyword of Intervertebral disc degeneration. Then, we analyzed the intersection gene set of ZKGCD targets and IDD-related genes through online analysis (http://bioinformatics.psb.ugent.be/webtools/Venn/) and obtained the intersection gene set containing 133 drug-disease targets, which are potential target genes of ZKGCD treatment for IDD (Figure 2 and Supplementary Table 3).

### 3.3. Construction of “Intersecting Genes-Active Components” Network

**Figure 3:**
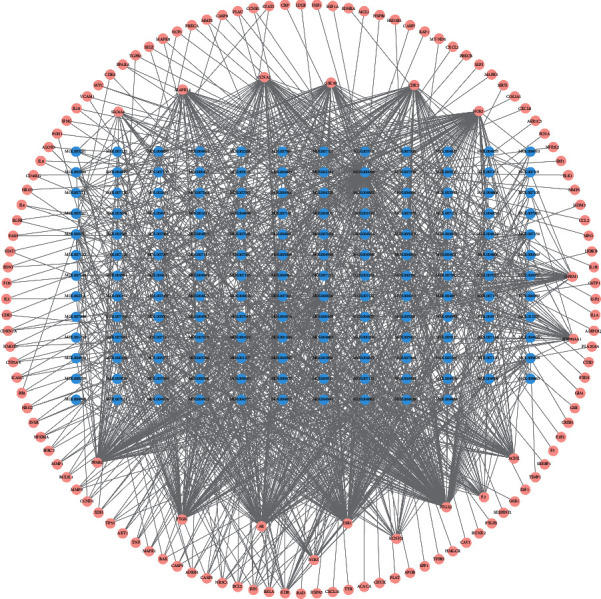
Intersecting genes-active components network. The pink circles represent intersecting genes between IDD and active ingredients in ZKGCD; the blue circles represent active ingredients related to the common genes; the lines between the two circles represent the interaction.

According to the relationship between drug targets and bioactive ingredients, we established the “intersection gene-active ingredient” network through Cytoscape software, which contains 1278 edges, 133 intersection genes, and 154 active ingredients (Figure 3). Then, we performed statistical analysis for this network structure and found that quercetin (Mol000098), luteolin (Mol000006), and kaempferol (Mol000422) were the top three degrees, which indicated that these three components correspond to the largest number of intersecting genes. Therefore, they may be the key components of ZKHCD for the treatment of IDD. The top 10 active ingredients were screened according to the degree value, as shown in Table 2.

### 3.4. Construction of Protein-Protein Interaction (PPI) Network and Key Genes Network

**Figure 4:**
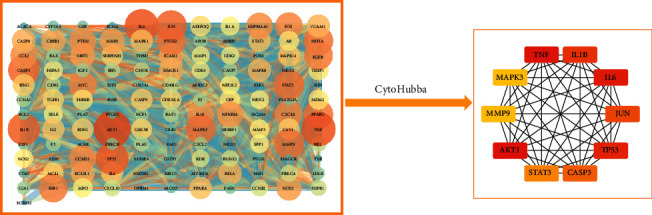
Protein-protein interaction (PPI) network and key gene network.

**Figure 5:**
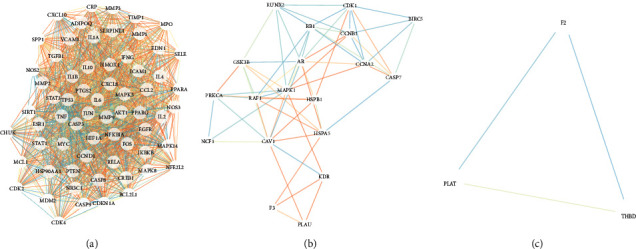
Three clusters of common genes by MCODE plugin in Cytoscape.

All 133 intersecting genes were imported into the STRING database to construct the PPI network and visualized it by Cytoscape (Figure 4). Then, based on the results of 12 topological algorithms, we selected the top 10 key genes based on degree values, namely AKT1, TNF, IL6, TP53, IL1B, JUN, CASP3, STAT3, MMP9, and MAPK3 (Figure 4). Additionally, a total of three clusters were identified in the PPI network by the cluster analysis function with the MCODE plugin (Figure 5, Table 3). We found that the first cluster contained 10 key genes, further confirming the credibility of key gene selection.

### 3.5. Construction of “Diseases-Key Genes-Active Ingredients-Herbs” Network

**Figure 6:**
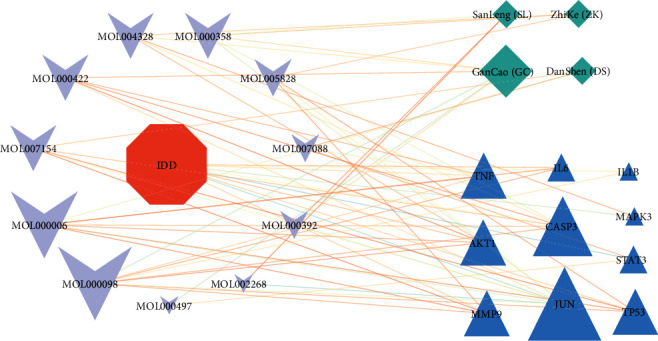
“IDD-key genes-active ingredients-herbs” network. Red pentagons represent diseases, dark blue triangles represent key genes, purple inverted triangles represent active ingredients associated with core genes, and light blue rectangles represent herbs. The size of each node represents the number of degrees.

To further elaborate the relationship between ZKGCD and IDD, we identified 11 bioactive ingredients and 4 herbs corresponding to 10 key genes, and the relationship among them is shown in Figure 6 (Supplementary Table 4). The 11 bioactive ingredients are quercetin (Mol000098), luteolin (Mol000006), kaempferol (Mol000422), beta-sitosterol (Mol000358), naringenin (Mol004328), nobiletin (Mol005828), formononetin (Mol000392), licochalcone A (Mol000497), cryptotanshinone (Mol007088), tanshinone IIA (Mol007154), and rhein (Mol002268). The 4 herbs are ZhiKe (ZK), GanCao (GC), DanShen (DS), and SanLeng (SL). Analyzing the “diseases-key genes-active ingredients-herbs” network revealed the highest degree of quercetin, the second degree of luteolin, and the third degree of kaempferol, which were consistent with the results of the “intersecting genes-active components” network analysis. Eight of the 11 bioactive ingredients are among the top 10 active ingredients in the “intersecting genes-active components” network.

### 3.6. Enrichment Analysis for GO Function and KEGG Pathway

**Figure 7:**
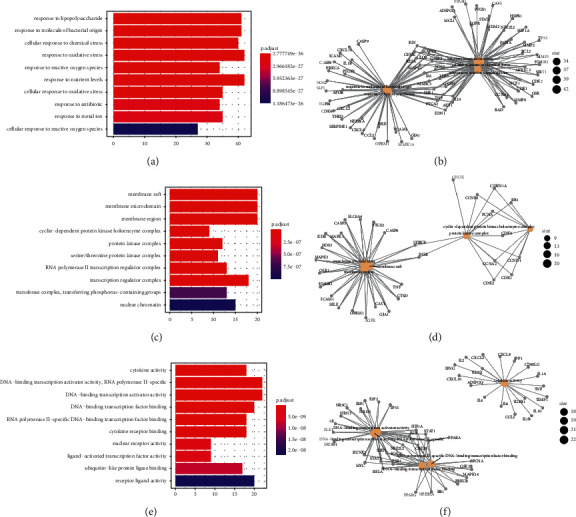
GO enrichment analysis of common genes. (a) Top 10 significantly enriched terms in biological processes (BPs). (b) Subnetwork showing the top five BP terms and related genes. (c) Top 10 significantly enriched terms in cellular components (CCs). (d) Subnetwork shows the top five CC terms and related genes. (e) Top 10 significantly enriched terms in molecular functions (MFs). (f) Subnetwork shows the top five MF terms and related genes.

Based on *R* platform, we conducted GO function and KEGG pathway enrichment analysis for 133 intersected genes. A total of 2610 results were obtained by GO functional enrichment analysis, including 2423 BPs, 50 CCs, and 137 MFs. Among them, biological processes are mainly related to response to oxidative stress, response to reactive oxygen species, cellular response to reactive oxygen species, reactive oxygen species metabolic process, and regulation of apoptotic signaling pathway. As for CCs, the results showed that it was mainly related to membrane raft, cyclin-dependent protein kinase holoenzyme complex, RNA polymerase II transcription regulator complex, nuclear chromatin, and vesicle lumen. The MFs are mainly related to cytokine activity, DNA-binding transcription activator activity, cytokine receptor binding, and receptor ligand activity.  Figure 7 exhibits the top 10 for each category, and details of the GO analysis results are listed in Supplementary Table 5.

**Figure 8:**
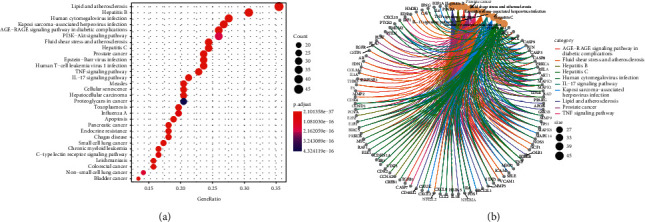
KEGG pathway enrichment analysis of common genes. (a) The 30 pathways with the lowest adjusted *p* values. The darker the color, the smaller the adjusted *p* value. The larger the circle, the greater the number of target genes in the term. (b) Subnetwork shows the top five KEGG pathways and related genes.

We used KEGG pathway enrichment analysis to further and comprehensively elaborate the potential mechanism of ZKGCD in delaying the IDD process. Finally, a total of 172 potential related pathways are enriched, mainly involving inflammation, apoptosis, senescence, and autophagy, which are specifically manifested as AGE-RAGE signaling pathway in diabetic complications (hsa04933), TNF signaling pathway (hsa04668), IL-17 signaling pathway (hsa04657), cellular senescence (hsa04218), apoptosis (hsa04210), and PI3K-Akt signaling pathway (hsa04151) (Table 4). Particularly, we visualized the first 30 pathways of enrichment results according to adjusted *P* values (Figure 8). Detailed information of the GO analysis results is listed in Supplementary Table 5.

### 3.7. Molecular Docking between Key Genes and Active Ingredients

**Figure 9:**
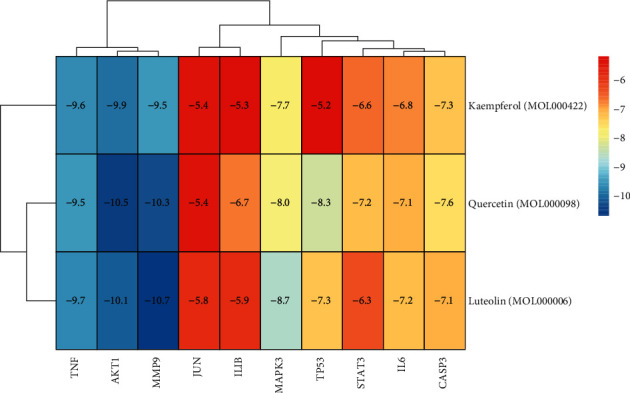
Heat map of binding energy between 10 core genes and top three active ingredients by molecular docking.

**Figure 10:**
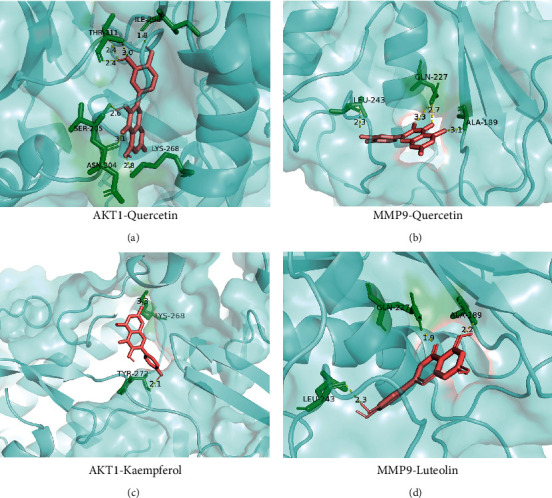
Four examples of conformations for some core compounds and key genes.

Binding energy is considered to be one of the key indicators to verify the stability of the conformation of the bound protein and active ingredient. At the same time, the stability of the conception increases as the binding energy decreases. For this reason, we performed molecular docking analysis on 10 key genes and the top 3 bioactive components, and the results are shown in Figure 9. The results of molecular docking showed that all binding energies were lower than −5.0 kcal/mol, which on the other hand reflected that ZKGCD acted through multiple targets in the treatment of IDD. In addition, we found that quercetin, the most important bioactive component of ZKGCD, obtained the highest binding energy of 10.5 kcal/mol for binding to the protein AKT1. Similarly, the highest binding energy of 9.9 kcal/mol was obtained for kaempferol binding to AKT1. However, for luteolin, the binding to MMP9 was required to obtain the highest binding energy of 10.7 kcal/mol. Moreover, we showed the structure of the interaction between each bioactive ingredient and the key protein with the strongest binding activity, whereas, for quercetin, we selected two key proteins (Figure 10).

## 4. Discussion

With the increasing intensity of modern work, the incidence of IDD is increasing. Currently, the generally recognized mechanisms of IDD may be related to the enrichment of inflammatory factors, senescence, and apoptosis of nucleus pulposus cells (NPC) and degradation of extracellular matrix (ECM). However, there is a lack of effective noninvasive treatment for IDD before it progresses to surgery. Single NSAIDs are mainly used to relieve painful symptoms, but it is accompanied by a series of side effects. Since herbs taken from nature have no or very few side effects, it has been used as a supplement to western medicine. ZKGCD, a traditional Chinese medicine compound, has been used to treat musculoskeletal diseases. However, the specific therapeutic mechanism of ZKGCD is not clear, which limited the application of ZKGCD in clinical practice. This study used network pharmacology theories, molecular docking techniques, high-throughput data analysis, and a series of related tools to visualize the specific relationships among drug active ingredients, key targets, important signaling pathways, and disease.

In this study, a total of 154 active ingredients were selected, among which quercetin (Mol000098), luteolin (Mol000006), and kaempferol (Mol000422) were identified as the core active ingredients. Quercetin is one of the main active components of licorice, which is a natural flavonoid with antioxidant and anti-inflammatory effects and widely exists in various plants. Several studies have shown that quercetin can delay IDD progression through multiple signaling pathways, mainly including (1) quercetin can prevent IDD by regulating p38 MAPK-mediated autophagy [30]; (2) quercetin can promote SIRT1-dependent autophagy to prevent IDD [31]; (3) QUE inhibits the expression of SASP and senescence phenotype in NPC and improves the progression of IDD through Nrf2/NF-*κ*B axis [32]. Luteolin, also a natural flavonoid, has anti-inflammatory and anticatabolic effects as its most important effects, which are opposed to the underlying mechanisms of IDD development. Therefore, it is reasonable that luteolin has a therapeutic effect on IDD. The most significant effect of kaempferol is anti-inflammatory, which has been shown to have beneficial effects on chronic inflammatory diseases, including IDD [33]. In addition, experimental studies have shown that kaempferol reduces inflammation mainly by increasing the levels of IL-10 and IL-6, which are anti-inflammatory and proinflammatory factors, respectively [34]. The major pathological features of IVDD are the elevated expression of inflammatory mediators, increased senescence and apoptosis of nucleus pulposus cells (NPCs), and degradation of the extracellular matrix [5, 6]. Therefore, regulating inflammation and oxidative stress is a crucial step in the treatment of IDD. Quercetin, luteolin, and kaempferol all have anti-inflammatory effects. We speculate that, on the one hand, the combination of multiple components may have produced a synergistic effect. On the other hand, other bioactive components may have promoted the antioxidant effect of luteolin. In addition, several studies have shown that the majority of herbal formulas with quercetin, kaempferol, and luteolin as the core bioactive components have regulated effects on inflammation, oxidative stress, and apoptosis, which is consistent with the potential mechanism of ZKGCD for IDD [35–38].

According to the ten key targets selected by the PPI network and topology algorithm, and the results of GO function enrichment and KEGG pathway enrichment analysis, the final results all mainly focused on the regulation of inflammatory response, oxidative stress response, reactive oxygen metabolism, and apoptotic signaling pathway. AKT1 is a serine/threonine protein kinase involved in a variety of biological processes. AKT activation depends on the PI3K pathway. Furthermore, studies have confirmed that the pathogenesis of disc lesions may be related to end-plate sclerosis, increased oxidative stress, and AGE/RAGE-mediated interactions. TNF and IL6, as typical inflammatory factors, are associated with our selected core active components and potential mechanisms (TNF signaling pathway and IL-17 signaling pathway) that are complementary to each other. The enrichment results also suggested that two pathways, including cellular senescence and apoptosis, may play a very important role in the treatment of IDD. On the one hand, senescence of NPC is a key factor in IDD, and delaying NPC senescence may be beneficial for alleviating IDD [39, 40]. On the other hand, endoplasmic reticulum (ER) stress and ECM degradation are important factors in the development of IDD. Autophagy can effectively repair ER stress and maintain ECM homeostasis [41]. Moreover, TP53 and CASP3, among the key targets, are mainly associated with induction of apoptosis and senescence, which further confirmed the potential of ZKGCD in treating IDD. However, we must acknowledge that the results of this study were confirmed by experiments in other studies, lacking our own in vivo experimental validation.

## 5. Conclusion

In conclusion, this study systematically elucidated the potential mechanisms of ZKGCD for the treatment of IDD based on a network pharmacology approach, molecular docking technique, and GO and KEGG enrichment analysis. The results indicated that quercetin (Mol000098), luteolin (Mol000006), and kaempferol (Mol000422) were the main bioactive components, which may alleviate the occurrence and development of IDD through the AGE-RAGE signaling pathway in diabetic complications, TNF signaling pathway, IL-17 signaling pathway, cellular senescence, apoptosis, and PI3K-Akt signaling pathway. This study demonstrated the characteristics of multicomponent, multitarget, and multipathway of ZKGCD and provided potential targets and a basis for the development of new drugs and experimental studies for the subsequent treatment of IDD.

## Figures and Tables

**Table 1:** Scientific names for all herbs in ZhiKe GanCao Decoction.

Pin Yin	Latin name
ZhiKe (ZK)	Aurantii Fructus
GanCao (GC)	Licorice
DangGui (DG)	Angelicae Sinensis Radix
DanShen (DS)	Radix Salvia
SanLeng (SL)	Sparganii Rhizome
Ezhu(EZ)	Curcumae Rhizome
QianNiuZi (QNZ)	Semen Pharbitidis

**Table 2:** Basic information of 11 active components in ZKGCD.

Molecule ID	Molecule name	PubChem CID	OB (%)	DL	Source (herb name)	Targeted key genes
MOL000098	Quercetin	5280343	46.43	0.28	GanCao	AKT1, MMP9, TNF, JUN, IL6, CASP3, TP53, IL1B
MOL000006	Luteolin	5280445	36.16	0.25	DanShen	AKT1, MMP9, TNF, JUN, IL6, CASP3, TP53
MOL000422	Kaempferol	5280863	41.88	0.24	GanCao	AKT1, TNF, JUN, CASP3
MOL004328	Naringenin	932	59.29	0.21	ZhiKe, GanCao	AKT1, MAPK3, CASP3
MOL000392	Formononetin	5280378	69.67	0.21	GanCao, SanLeng	JUN
MOL005828	Nobiletin	72344	61.67	0.52	ZhiKe	MMP9, JUN, TP53
MOL007154	Tanshinone IIA	164676	49.89	0.40	DanShen	MMP9, JUN, CASP3, TP53
MOL000497	Licochalcone A	5318998	40.79	0.29	GanCao	STAT3
MOL000354	Isorhamnetin	5281654	49.60	0.31	GanCao	/
MOL004373	Anhydroicaritin	44259058	45.41	0.44	QianNiuZi	/

**Table 3:** Cluster information of the protein-protein interaction (PPI) network for common genes.

Cluster	Score	Nodes	Edges	Gene names
1	50.156	65	1605	MMP2, TGFB1, SERPINE1, MPO, CCL2, CDK4, MYC, IL2, MDM2, IFNG, IL4, NR3C1, CRP, CDK2, MAPK3^*∗*^, IL1A, STAT3^*∗*^, ICAM1, TP53^*∗*^, VCAM1, NOS3, MMP3, BCL2L1, CXCL8, SELE, CXCL10, JUN^*∗*^, MMP9^*∗*^, MMP1, EDN1, SPP1, NOS2, MAPK8, CASP9, HSP90AA1, SIRT1, IKBKB, HIF1A, PPARG, ESR1, IL10, CASP8, STAT1, PTEN, EGFR, PPARA, ADIPOQ, MCL1, AKT1^*∗*^, CHUK, IL1B^*∗*^, FOS, NFKBIA, PTGS2, NFE2L2, CREB1, TNF^*∗*^, CCND1, CASP3^*∗*^, RELA, IL6^*∗*^, MAPK14, CDKN1A, HMOX1, TIMP1
2	7	19	63	NCF1, HSPB1, RAF1, AR, F3, PLAU, CCNB1, BIRC5, MAPK1, GSK3B, CAV1, CDK1, KDR, PRKCA, RB1, RUNX2, CCNA2, CASP7, HSPA5
3	3	3	3	F2, THBD, PLAT

*∗*Core genes are highlighted in red.

**Table 4:** The main related pathways for IDD are in the top 30.

ID	Description	p. adjust	Count
hsa04933	AGE-RAGE signaling pathway in diabetic complications	3.36E-34	30
hsa04668	TNF signaling pathway	1.53E-26	29
hsa04657	IL-17 signaling pathway	4.17E-26	27
hsa04218	Cellular senescence	5.35E-19	26
Hsa04210	Apoptosis	3.71E-18	24
Hsa04151	PI3K-Akt signaling pathway	2.00E-16	33

## Data Availability

The data used to support the findings of this study are included within the article.

## Supplementary Materials

Supplementary Materials Supplementary Table 1: ingredients of each herb contained in ZKGCD (OB ≥ 30%, DL ≥ 0.18). Supplementary Table 2: potential targets related to active ingredients in ZKGCD. Supplementary Table 3: intersection genes and corresponding active ingredients. Supplementary Table 4: relationship among key genes, active ingredients, and herbs. Supplementary Table 5: detailed information of GO and KEGG enrichment analysis for common targets.
