# Supplementary figures and images for: Systematic Elaboration of the Pharmacological Targets and Potential Mechanisms of ZhiKe GanCao Decoction for Preventing and Delaying Intervertebral Disc Degeneration
Source: Evid Based Complement Alternat Med. 2022 Apr 22;2022:8786052. doi: 10.1155/2022/8786052 (PMC9054440; doi:10.1155/2022/8786052)

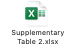
 Please double-click to view the table.

Supplement: Supplementary Materials — Supplementary Table 1: ingredients of each herb contained in ZKGCD (OB ≥ 30%, DL ≥ 0.18). Supplementary Table 2: potential targets related to active ingredients in ZKGCD. Supplementary Table 3: intersection genes and corresponding active ingredients. Supplementary Table 4: relationship among key genes, active ingredients, and herbs. Supplementary Table 5: detailed information of GO and KEGG enrichment analysis for common targets. [file 8786052.f1.zip › 8786052.f1/Supplementary Table 2 (1).docx]

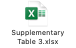
 Please double-click to view the table.

Supplement: Supplementary Materials — Supplementary Table 1: ingredients of each herb contained in ZKGCD (OB ≥ 30%, DL ≥ 0.18). Supplementary Table 2: potential targets related to active ingredients in ZKGCD. Supplementary Table 3: intersection genes and corresponding active ingredients. Supplementary Table 4: relationship among key genes, active ingredients, and herbs. Supplementary Table 5: detailed information of GO and KEGG enrichment analysis for common targets. [file 8786052.f1.zip › 8786052.f1/Supplementary Table 3 (1).docx]

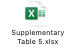
 Please double-click to view the table.

Supplement: Supplementary Materials — Supplementary Table 1: ingredients of each herb contained in ZKGCD (OB ≥ 30%, DL ≥ 0.18). Supplementary Table 2: potential targets related to active ingredients in ZKGCD. Supplementary Table 3: intersection genes and corresponding active ingredients. Supplementary Table 4: relationship among key genes, active ingredients, and herbs. Supplementary Table 5: detailed information of GO and KEGG enrichment analysis for common targets. [file 8786052.f1.zip › 8786052.f1/Supplementary Table 5 (1).docx]
